# Are three-dimensional patient-specific cutting guides for open wedge high tibial osteotomy accurate? An in vitro study

**DOI:** 10.1186/s13018-018-0872-4

**Published:** 2018-07-09

**Authors:** Mathias Donnez, Matthieu Ollivier, Maxime Munier, Philippe Berton, Jean-Pierre Podgorski, Patrick Chabrand, Sébastien Parratte

**Affiliations:** 1 Aix Marseille Univ, CNRS, ISM, Marseille, France; 2 Aix Marseille Univ, APHM, CNRS, ISM, Sainte-Marguerite Hospital, Institute for Locomotion, Department of Orthopaedics and Traumatology, Marseille, France; 3 Newclip Technics, Haute-Goulaine, France

**Keywords:** Knee surgery, Osteoarthrosis, Medial gonarthrosis, Osteotomy, Open wedge high tibial osteotomy, Patient-specific, Accuracy, Tibial slope correction

## Abstract

**Background:**

The aim of this in vitro study was to assess the accuracy of three-dimensional patient-specific cutting guides for open wedge high tibial osteotomy (OWHTO) to provide the planned correction in both frontal and sagittal planes.

**Methods:**

Ten cadaveric tibias underwent OWHTO performed using a patient-specific cutting guide based on 3D preoperative planning. An initial CT scan of the tibias was performed, and after segmentation, 3D geometrical models of the pre-OWHTO tibias were obtained. Reference planes were defined, and OWHTO virtually planned to then design patient-specific cutting guides. OWHTO were performed using the patient-specific cutting guides. The patient-specific cutting guide controls the cut and the correction of the OWHTO in both planes. 3D models of post-OWHTO tibias were created after a postoperative CT scan. Geometrical post-OWHTO 3D models were superimposed on pre-OWHTO 3D models. Mechanical medial proximal tibial angle (mMPTA) in the frontal plane and posterior tibial slope (PTS) in the sagittal plane were compared between planned-OWHTO and post-OWHTO 3D reconstructions relative to the pre-OWHTO reference planes and axis. Pearson’s and Lin’s correlation tests were performed to assess precision and accuracy of patient-specific cutting guides.

**Results:**

The mean difference between post-OWHTO and planned-OWHTO was 0.2° (max 0.5°, SD 0.3°) in the frontal plane and − 0.1° (max 0.8°, SD 0.5°) in the sagittal plane. Statistically significant correlations were found between the planned-OWHTO and post-OWHTO configurations for the mMPTA (*p* < 0.0001) and PTS (*p* < 0.0001) measurements, and the bias correction factor was 0.99 in both planes.

**Conclusions:**

3D patient-specific cutting guides for OWHTO-based 3D virtual planning is a reliable and accurate method of achieving multiplanar correction in both frontal and sagittal planes.

## Background

Open wedge high tibial osteotomy (OWHTO) has been described as an efficient conservative surgical treatment preserving the bone stock for patients with moderate medial gonarthrosis and lower leg malalignment [1, 2]. The objective of the OWHTO is to correct lower leg malalignment in both the frontal and sagittal tibial planes to limit the overload of the medial compartment. Accurate correction is essential to its success as under correction leads to persistent pain and over correction to functional limitations [3, 4].

Different methods and instrumentations have been developed to help the surgeon to achieve the planned correction [3, 5–7].

Conventional methods use standard instrumentation to open the osteotomy [5, 6]. Frontal correction can then be managed intraoperatively by measuring the opening angle or opening gap and comparing it to the preoperative plan [8–10], or by using a radiopaque cable under fluoroscopy to control lower limb alignment [9, 11–13]. The control of the correction for both the frontal and the sagittal planes at the same time with standard instrumentations remains challenging [14–16]. Computer-assisted surgery (CAS) for OWHTO was validated experimentally in vitro by Hankemeier et al. [7] and subsequently used in clinical studies [3, 17–21]. CAS allows real-time control of the correction. All these studies reported better accuracy and reliability for CAS than for conventional or cable methods, but with increased surgical time and a control of the global alignment of the limb but not of tibia only and not the posterior tibial slope (PTS).

While frontal plane correction is most often described for osteotomy management, PTS management in the sagittal plane is essential to preserve biomechanics [22–24]. Song et al. [19] reported that PTS is unchanged if the anterior opening is equal to 67% of the medial opening. However, this evaluation is complex to perform during surgery, and patient-specific instruments may help the surgeon to manage both the sagittal and the frontal plane correction during surgery [25, 26].

Using an experimental setup, three-dimensional patient-specific cutting guides were reported to be more accurate than free-hand technique to perform an osteotomy cut and drill in a synthetic bone [27]. The use of patient-specific cutting guides for OWHTO was also reported recently in three studies realized on small series of patients [25, 26, 28]. All three clinical studies reported good accuracy and reliability of the procedure. Planning procedures for OWHTO were carried out either on 2D long-leg radiographs [28] or from a 3D model [25, 26]. Pérez-Mañanes et al. used CT images to obtain the 3D tibial surface and to position two K-wires to lead the cut, and the correction was planned using two additional wedges.

To avoid the disappointing clinical results observed with patient-specific instrumentation for total knee arthroplasty, we wanted to evaluate in vitro this new technique. After developing a specific patient-specific guide to control both the cut and the correction adapted for a specific OWHTO plate, it was our aim to evaluate the accuracy of the system in an in vitro CT scan-controlled study. Our hypothesis was that the patient-specific cutting guide for OWHTO can provide an accurate correction in both the frontal and sagittal planes.

## Methods

### Study design

In this in vitro study, ten frozen cadaveric specimens’ tibias (eight females and two males aged from 70 to 99, average age 88, 5 right sides) were obtained from our Department of Anatomy at the Aix-Marseille University School of Medicine (Table 1). The subjects were all preserved in Winckler liquid [29, 30]. All soft tissues were removed, except the patellar tendon insertion.

**Table 1:** Specimen description

Specimen	Gender	Age	Side	Pre-OWHTO mMPTA (°)	Pre-OWHTO PTS (°)
G068	F	99	R	89.6	7.9
G131	F	78	R	88.5	2.1
G059	F	95	L	84.2	5.3
G141	F	99	L	88.0	2.4
G115	M	88	R	86.9	4.7
G111	F	89	R	88.5	2.9
G119	M	90	R	88.1	4.5
G113	F	84	L	89.5	7.6
G136	F	90	L	90.3	4.2
G117	F	70	L	83.6	9.8

Demographic data, pre-OWHTO measurements

### OWHTO preoperative planning

**Fig. 1:**
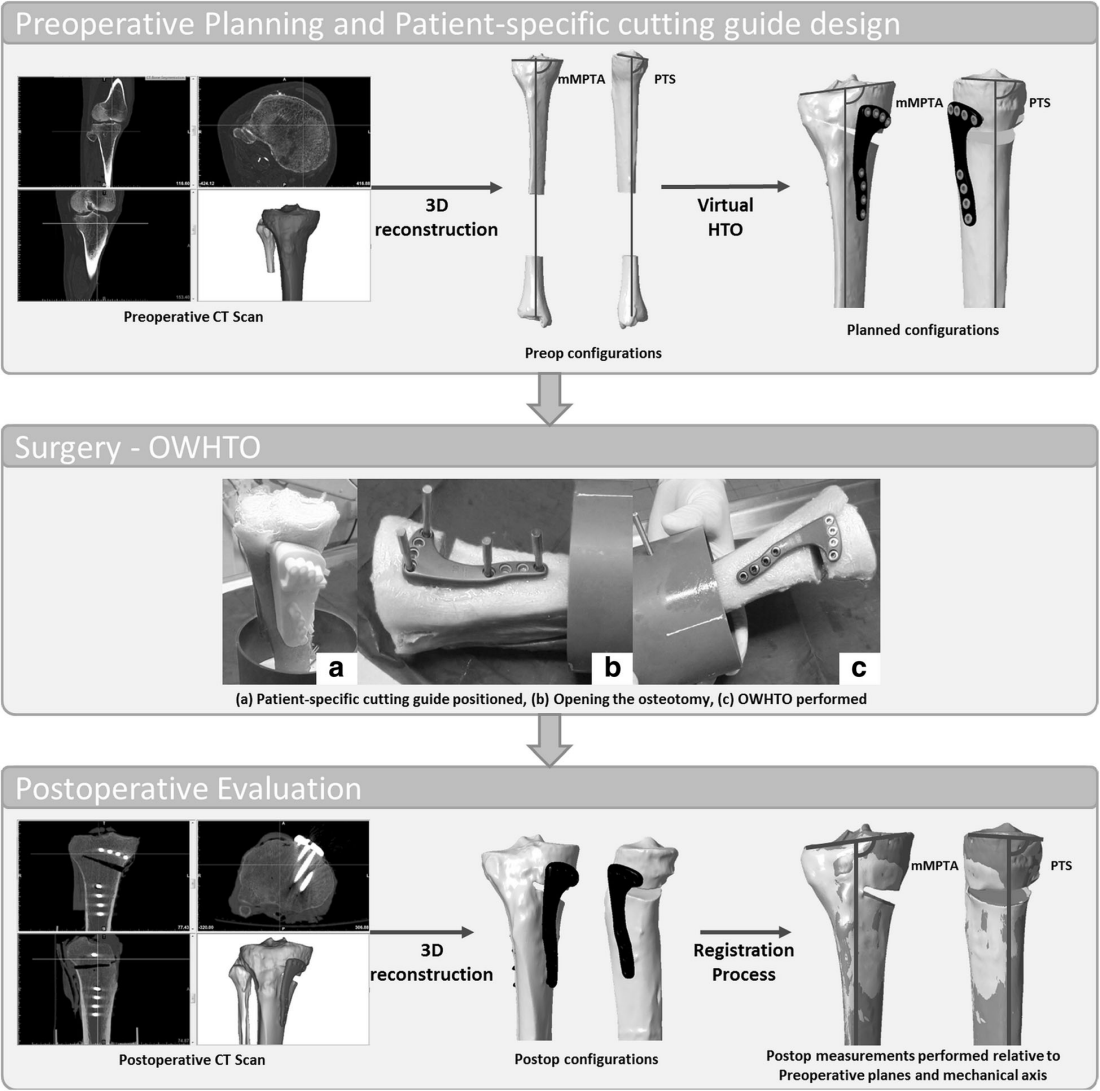
Overview of the experimental protocol. All measurements are performed in the preoperative reference planes and relative to the preoperative mechanical axis

All specimens were scanned using a standardized CT scan protocol (Discovery 710, GE Medical System, CERIMED, Marseille, France). The following acquisition parameters were used both prior and after the HTO: 120 kV, 400 mA, and 0.625-mm-thick slices. DICOM images were imported into Mimics 17.0 software (Materialise®, Leuven, Belgium), and 3D geometrical models of the tibias were created (Pre-OWHTO configurations, Fig. 1).

**Fig. 2:**
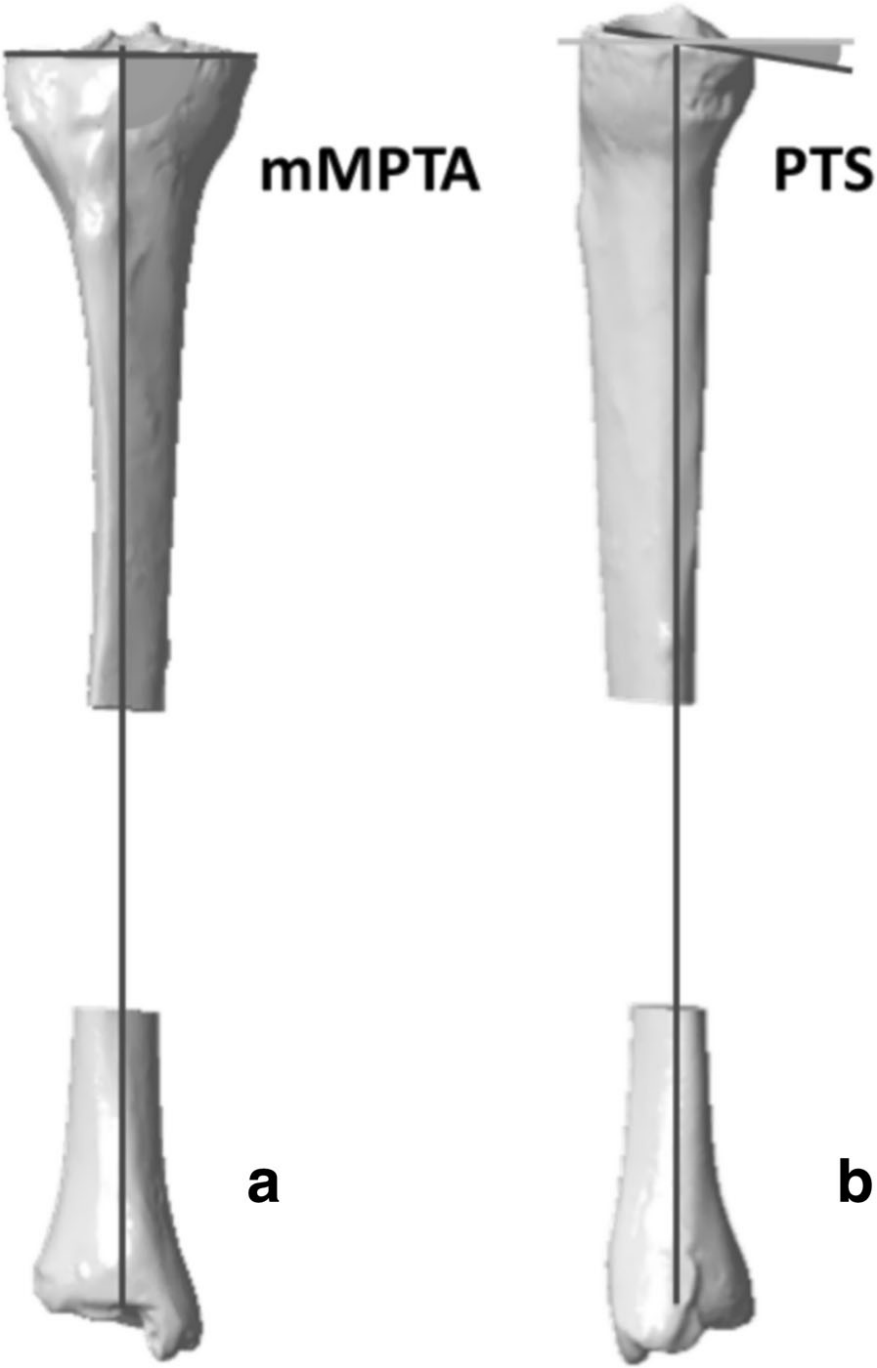
Angle measurements on 3D models. **a** mMPTA measures the medial angle between the tibial mechanical axis and the tangent mediolateral line of the tibial plateau in the frontal plane. **b** PTS is the posterior angle between the orthogonal line to the mechanical axis and the anteroposterior tangent line of the medial plateau in the sagittal plane

Anatomical landmarks, anatomical reference planes, and the mechanical axis of the tibia were defined on the 3D models according to Lee et al. [31]. Preoperative mechanical medial proximal tibial angle (mMPTA) and medial PTS were measured (Fig. 2). mMPTA measures the varus deformation of the tibia. PTS measures the sagittal orientation of the proximal tibia. For each specimen, a correction for proximal tibial bony deformity, when present, was determined in both the frontal and the sagittal planes. For specimens with optimal tibial mechanical alignment, a random correction was applied. Each preoperative 3D tibia model was imported to a specially designed 3D planning tool for OWHTO. A cutting plane was positioned, and OWHTO was simulated with respect to the frontal and sagittal plane corrections previously determined (planned-OWHTO configurations, Fig. 1). The Activmotion-2 plate (Newclip Technics®, Haute-Goulaine, France) was positioned on the anteromedial surface of the tibia following the manufacturer’s recommendations. The plate contains four locking screws above and four locking screws below the osteotomy cut. Then, a patient-specific cutting guide was designed for each tibia based on the OWHTO simulation. Patient-specific cutting guides took into account the tibial anatomy, the position of the cutting plane, the amount of correction planned in all planes, and the plate location. All patient-specific cutting guides were 3D printed.

### OWHTO

Specimens were thawed overnight. All OWHTO were performed by one surgeon (MM) using the patient-specific cutting guides [26]. Then, the same CT scan protocol was used to assess the tibias after OWHTO. Post-OWHTO 3D geometrical models were created with Mimics 17.0 software (post-OWHTO configurations, Fig. 1).

### Registration process

**Fig. 3:**
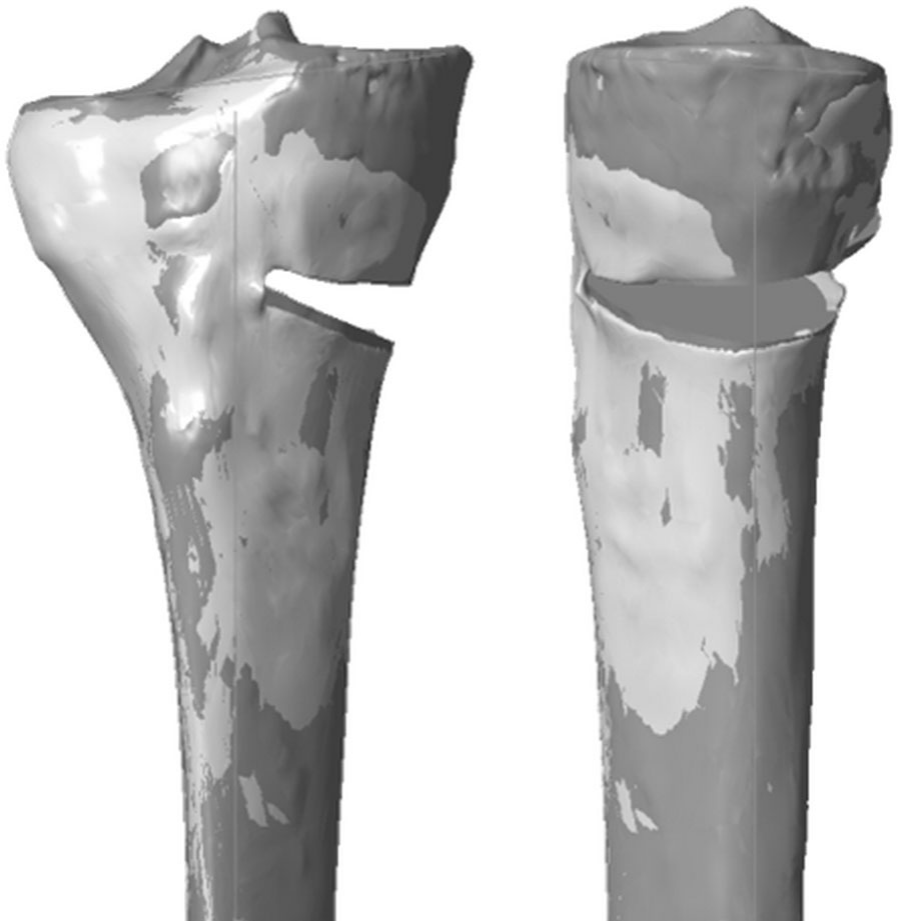
The post-OWHTO model was registered on the planned-OWHTO model

For each specimen, the distal part of the post-OWHTO tibia was superimposed on the distal part of the pre-OWHTO tibia using an iterative closest point (ICP) algorithm (Fig. 3). This registration process ensured that measurement references for all models of each tibia were the same. Then, mMPTA and PTS were measured on both the planned-OWHTO and the post-OWHTO configuration. For maximum reproducibility of measurements, both planned and postoperative angles were measured in the frontal and sagittal pre-OWHTO planes relative to the pre-OWHTO tibial mechanical axis.

Planned-OWHTO and post-OWHTO configurations were compared to assess the precision and the accuracy provided by the patient-specific cutting guides for OWHTO.

### Statistical analysis

Statistical analysis was performed between the planned-OWHTO and the post-OWHTO mMPTA, and between the planned-OWHTO and the post-OWHTO PTS. Correlation analyses were performed using Pearson’s correlation test to assess the precision reached by the patient-specific cutting guides. Accuracy was assessed by the calculation of the bias correction factor (*C*_b_) given by Lin’s correlation test [32].

Significance was considered at *p* < 0.05. The 95% confidence intervals (CI) were presented. Statistical analysis was performed on R software.

## Results

The mean pre-OWHTO mMPTA was 87.7° (SD 2.2°), and the mean pre-OWHTO PTS was 5.1° (SD 2.6°). A mean 7.3° (SD 1.3°) correction in the frontal plane and a 3.3° (SD 2.7°) correction in the sagittal plane were thus planned. For all specimens, the positioning of the patient-specific cutting guides on the bone was possible in accordance with the planning. All patient-specific cutting guides fitted the tibial surface, so all OWHTO were performed as planned.

Differences between the post-OWHTO and planned-OWHTO configurations were calculated. The mean difference between the post-OWHTO and planned-OWHTO configurations was 0.2° (from − 0.3° to 0.5°, SD 0.3°) in the frontal plane, and − 0.1° (from − 0.7° to 0.8°, SD 0.5°) in the sagittal plane (Table 2).

**Table 2:** Angular measurements

	Pre-OWHTO		Corrections		Planned-OWHTO		Post-OWHTO		Planned-OWHTO vs post-OWHTO	
Specimen	mMPTA (°)	PTS (°)	mMPTA (°)	PTS (°)	mMPTA (°)	PTS (°)	mMPTA (°)	PTS (°)	mMPTA (°)	PTS (°)
G068	89.6	7.9	8	5	97.3	3.0	97.0	3.5	0.3	− 0.5
G131	88.5	2.1	6	0	94.5	2.1	94.7	2.3	− 0.2	− 0.2
G059	84.2	5.3	8	0	92.9	5.2	92.9	4.4	0.0	0.8
G141	88.0	2.4	9	3	97.0	− 0.7	96.6	− 0.6	0.4	− 0.1
G115	86.9	4.7	8	4	95.2	0.7	94.7	1.2	0.5	− 0.5
G111	88.5	2.9	7	3	95.1	− 0.1	95.2	0.3	− 0.1	− 0.4
G119	88.1	4.5	6	4	94.1	0.6	93.6	0.9	0.5	− 0.3
G113	89.5	7.6	5	6	94.1	1.7	93.7	1.1	0.4	0.6
G136	90.3	4.2	7	0	97.3	4.1	97.6	3.9	− 0.3	0.2
G117	83.6	9.8	9	8	92.2	1.9	91.8	2.6	0.4	− 0.7

Pre-OWHTO measurements, planned corrections, planned-OWHTO measurements, post-OWHTO measurements, and absolute difference between planned-OWHTO and post-OWHTO configurations

**Fig. 4:**
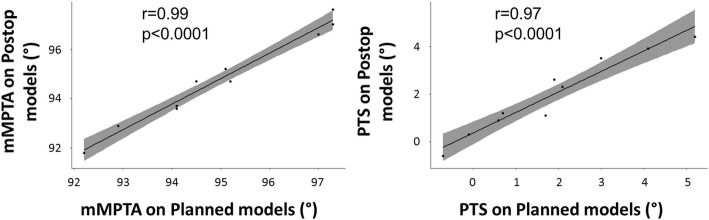
Correlations between planned-OWHTO and postop-OWHTO models for mMPTA (**a**) and PTS (**b**)

According to Pearson’s correlation tests, a statistically significant correlation was found between the planned-OWHTO and post-OWHTO configurations, for the mMPTA measurements with a correlation coefficient of 0.99 (95% CI, 94–99%, *p* < 0.0001), and for the PTS measurements with a correlation coefficient of 0.97 (95% CI, 86–99%, *p* < 0.0001) (Fig. 4).

Bias coefficient *C*_b_ was 0.99 in both the frontal and sagittal planes (Table 3).

**Table 3:** Statistical analysis results

	Pearson’s correlation coefficient	*C*_b_
mMPTA	0.99 (95% CI, 94–99%)	0.99
PTS	0.97 (95% CI, 86–99%)	0.99

Pearson’s correlation coefficient and bias correction factor *C*_b_ between planned-OWHTO and post-OWHTO configurations

## Discussion

OWHTO success remains on accurate correction in order to avoid persistent pain or functional limitations [3, 4]. This in vitro study aimed to investigate the accuracy in both the frontal and sagittal planes provided by patient-specific cutting guides with respect to 3D planning of OWHTO. The hypothesis that patient-specific cutting guides can provide the planned correction in both the frontal and sagittal planes was confirmed in this CT scan-controlled in vitro study.

In this patient-specific procedure for OWHTO, bone deformation angles were measured with respect to the anatomical reference planes determined using the procedure of Lee et al. [31] in order to mimic clinical practice. Clinical studies report several methods of finding anatomical reference points and measuring deformation angles [25]. Seeking to achieve maximum reliability and to measure only the correction provided by the patient-specific cutting guide, all measurements were performed relative to the same axis and in the same planes for the pre-OWHTO, planned-OWHTO, and post-OWHTO configurations. Unlike Munier et al., who performed measurements separately on the preoperative and postoperative 3D configurations, and then compared how far the two configurations differed from the planned correction, we performed our measurements relative to the preoperative mechanical axis. This was made possible by the registration process we used to superimpose the post-OWHTO configuration on the planned-OWHTO configuration. As our objective was to assess the amount of correction, it was vital to keep the reference definition the same for both planned-OWHTO and post-OWHTO configurations.

Intraoperative methods to control correction are varied, have limited accuracy, and mainly focus on the frontal plane correction. In their review of the literature, Van Den Bempt et al. reported ranges of accuracy in the frontal plane from several clinical studies [4]. For conventional methods, the mean amplitude of the range of accuracy was 5.6° (from 4° to 8°) [33–37], whereas using the CAS method, it was 5.5° (from 4° to 7°) [10, 17, 38–41]. Irrespective of the range of accuracy chosen by the authors, the literature reports a mean 32% of outlier patients when authors used conventional methods [9, 10, 17, 21, 34–37, 40, 42–45] and 22% when they used CAS [10, 17, 21, 38–42, 44, 46]. Some in vitro studies find CAS to be accurate for OWHTO. On a single synthetic bone and with a statistical model, Keppler et al. [47] evaluated a mean error of 0.7° in the frontal plane between the postoperative results and the target. Wang et al. [48] performed OWHTO with several amounts of correction in a synthetic bone using CAS and reported 0.4° accuracy in the frontal plane. Both authors validated their experimental work in a preliminary clinical study, on five and four patients. They both found a mean error of 1° in the frontal plane. On a single cadaveric specimen, Lützner et al. [49] evaluated the accuracy of CAS to measure lower limb alignment. They found a mean error of 0.6° in the frontal plane. Hankemeier et al. [7] performed OWHTO on 20 legs randomly assigned to CAS or a conventional method. They reported better accuracy and less variability than with a cable method. Among clinical studies using patient-specific cutting guides, Menetrey et al. [20] evaluated the frontal plane correction, based on 2D measurements for planning. Their patient-specific cutting guide only incorporates the cutting plane position, the correction being guided by two additional wedges. They found 0.5° accuracy (from 0° to 1.2°) in the frontal plane. Patient-specific cutting guides containing both the cutting plane planning and the correction were used in two other studies [19, 21]. In one, Munier et al. performed a postoperative 3D evaluation of their patients. Overall, they found similar accuracy for both 2D and 3D measurements by reproducing the measurement protocol on the postoperative model [26] around 0.0° (from − 1.7° to 1.8°, SD 1.1°) in the frontal plane. In the other study, Victor and Premanathan [25] reported a mean difference of 0.1° (from − 1° to 1°, SD − 0.1°) in the frontal plane.

Controlling the sagittal plane correction is essential to preserve knee biomechanics [23–25], but conventional methods have limited accuracy, remaining on gap measurements [15, 19]. Using CAS on synthetic bones, a mean error of 0.9° [47] and a 0.5° accuracy [48] were reported in the sagittal plane. Using patient-specific cutting guides, an accuracy around 0.3° (from − 2° to 3.2°, SD 1.4°) [26] and a mean difference of − 0.1° (from − 3° to 2°, SD 1.2°) [25] in the sagittal plane were reported.

In this study, the patient-specific OWHTO procedure includes 3D preoperative planning of the surgery and the design of a patient-specific cutting guide which takes into account the tibial anatomy, the position of the cutting plane, the amount of correction planned in all planes, and the plate location. The mean differences between the planned-OWHTO and post-OWHTO models are 0.2° (from − 0.3° to 0.5°, SD 0.3°) in the frontal plane and − 0.1° (from − 0.7° to 0.8°, SD 0.5°) in the sagittal plane. OWHTO outcomes are compared with the planning by superimposing the post-HTO 3D reconstruction on the planning 3D model. This enabled to assess the accuracy of patient-specific cutting guides. Findings of the present study suggest that PTS is managed with accuracy, which is important for the management of cruciate ligament balance [23, 25].

One limitation of our study is the small number of specimens used. Moreover, not all specimens had a sufficient varus deformation in their proximal tibia to be considered as candidates for OWHTO. However, different degrees of correction consistent with clinical practice were planned in both the frontal and sagittal planes. We also departed from clinical practice in performing all the measurements on 3D models. This was possible thanks to the cadaveric nature of the specimens. This enabled us to assess precision and accuracy by comparing post-OWHTO and planned-OWHTO 3D models, thereby avoiding measurement errors related to 2D radiography. The preoperative CT scan required for this procedure could be another limitation for direct clinical application, not being necessary in conventional or CAS procedures. However, Menetrey et al. [20] reported that the patient-specific cutting guide reduced the use of intraoperative fluoroscopy from 55 images on average (range, 41–73) in conventional methods to 8 (range, 6–14), as well as requiring less surgical time.

## Conclusion

OWHTO is demanding surgery, and accuracy of the correction in both frontal and sagittal planes is essential to its success. This study shows that combining 3D planning with patient-specific cutting guides for OWHTO is a reliable and accurate method of achieving multiplanar correction in both the frontal and the sagittal planes. Further randomized clinical studies should be carried out to validate these experimental results and evaluate the risk-benefit ratio of the preoperative CT scan, and the reduction in surgical time.
